# Enhanced Recovery After Surgery (ERAS) and Surgical Site Infections (SSIs)

**DOI:** 10.3390/antibiotics15060602

**Published:** 2026-06-12

**Authors:** Marco Catarci, Luca Pellegrino, Paolo Ciano, Sara Salomone, Michele Benedetti, Felice Borghi

**Affiliations:** 1 General Surgery Unit, Sandro Pertini Hospital, ASL Roma 2, Via dei Monti Tiburtini, 385, 00157 Rome, Italy; paolo.ciano@aslroma2.it (P.C.); michele.benedetti@aslroma2.it (M.B.); 2 Department of Surgery, Candiolo Cancer Institute, FPO-IRCCS, 10060 Turin, Italy; luca.pellegrino@ircc.it (L.P.); sara.salomone@ircc.it (S.S.); felice.borghi@ircc.it (F.B.)

**Keywords:** ERAS, surgical site infections, perioperative infections, infection prevention, perioperative care, ERAS and SSI

## Abstract

Enhanced Recovery After Surgery (ERAS^®^) is a multimodal perioperative framework designed to mitigate the physiological stress response to major surgery. While ERAS protocols consistently reduce length of hospital stay, overall complication rates, and healthcare costs compared to conventional care, their specific impact on surgical site infections (SSIs) remains poorly defined. This review explores the potential synergistic benefits of integrating ERAS protocols with established infection prevention bundles. By evaluating the current clinical evidence, we analyze how the co-implementation of these two evidence-based strategies can collectively reduce the incidence of SSIs.

## 1. Introduction

### 1.1. Definition and Origins of ERAS

**Figure 1:**
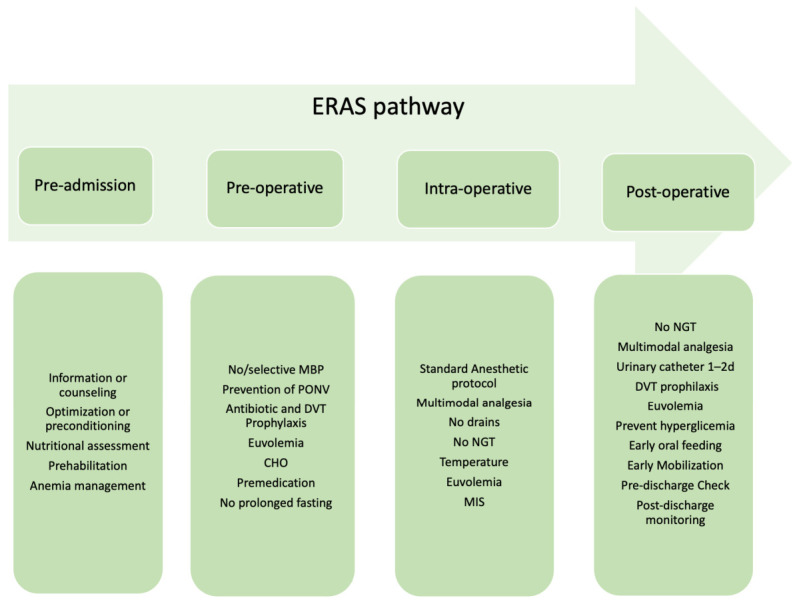
ERAS items for colorectal surgery. [MBP: mechanical bowel preparation. PONV: post-operative nausea and vomiting; DVT: deep vein thrombosis; CHO: carbohydrate overloading; NGT: nasogastric tube; MIS: minimally invasive surgery].

Enhanced Recovery After Surgery (ERAS^®^) is a multimodal perioperative care pathway designed to attenuate the surgical stress response in patients undergoing major procedures. The core objectives of ERAS pathways encompass active patient education, shared decision-making, maintenance of euvolemia, optimization of analgesia, and accelerated functional recovery. Originally conceptualized as “fast-track surgery” by Henrik Kehlet in the 1990s for colonic procedures, the protocol integrates discrete, evidence-based elements that are individually validated to improve postoperative outcomes [1]. When these elements—categorized into preadmission, preoperative, intraoperative, and postoperative phases—are implemented collectively, they exert a synergistic effect that surpasses conventional perioperative care. Given that ERAS involves various healthcare specialties, a multidisciplinary approach is essential. Owing to the program’s inherent complexity, teams must engage in continuous clinical audits of the processes and outcomes to ensure iterative improvements and sustained effectiveness. In 2010, the ERAS^®^ Society was established in Stockholm as a non-profit medical organization. Its mission is to advance perioperative care through research, education, and the implementation of evidence-based guidelines, which are regularly updated and hosted on its official website (https://erassociety.org, accessed on 15 March 2026). Figure 1 delineates the specific ERAS components commonly utilized in colorectal surgery.

### 1.2. Surgical Site Infections (SSIs)

Surgical site infections (SSIs) represent the most frequent healthcare-associated complication following major surgery, occurring in up to 5% of all surgical cases. Their clinical and systemic consequences are profound, manifesting as a 2- to 11-fold increase in mortality risk, prolonged hospitalizations, and elevated readmission rates [2]. Beyond these macroscopic metrics, SSIs severely compromise patient-reported outcomes by exacerbating postoperative anxiety and reducing health-related quality of life, while concurrently imposing a substantial economic burden on healthcare infrastructure [3].

### 1.3. Adherence to the ERAS Protocol and Outcomes

Quantifying the distinct clinical benefits of ERAS pathways via traditional comparative methodologies presents unique challenges. Double-blinding is rarely feasible in surgical delivery science, and the absolute number of protocol items implemented across heterogeneous trial designs varies widely, confounding uniformity in randomized controlled trials. Nevertheless, meta-analytic data from colorectal surgery (CRS) demonstrate robust improvements over conventional care, including a 2.0- to 2.5-day reduction in length of stay (LOS) and a 30% to 50% decrease in overall complication rates [4]. Furthermore, data from multiple large-scale prospective registries [5,6,7,8,9,10] demonstrate a direct dose–response relationship between pathway adherence and clinical efficacy. Current literature establishes a compliance threshold of 70% to 80% as the critical benchmark necessary to unlock significant clinical dividends, including reductions in major morbidity and mortality [6,7,8,9], a decrease in failure-to-rescue rates [10], and improved long-term overall survival [11].

### 1.4. Impact of ERAS on SSIs

**Figure 2:**
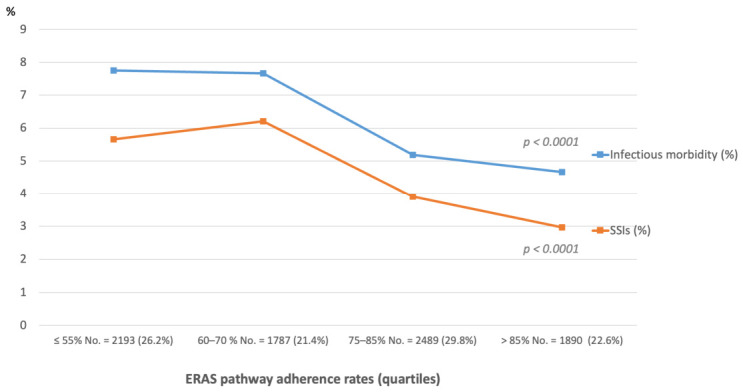
Association between ERAS protocol compliance quartiles and infectious complication rates in Italy. Data synthesized from the prospective multicenter iCral cohort [9].

The specific efficacy of ERAS protocols in mitigating SSIs remains a subject of active debate within the surgical community. Certain investigations [12,13] found no independent reduction in SSI incidence attributable to ERAS, identifying minimally invasive surgery (MIS) as a more dominant protective cofactor [12]. Conversely, the multicenter POWER study—enrolling 2084 prospective colorectal patients in Spain—demonstrated that institutions maintaining high ERAS adherence (greater than 77%) experienced significantly lower infectious complication rates than those with low compliance (less than 54%) [7]. Similarly, the iCral study in Italy, which evaluated 8359 prospective colorectal patients, revealed a stepwise reduction in both independent SSIs and composite infectious endpoints (the aggregate of surgical site, pulmonary, and urinary tract infections) across increasing quartiles of protocol adherence [9] (Figure 2). Beyond colorectal surgery, ERAS principles have been adapted across diverse surgical disciplines, including upper gastrointestinal, bariatric, hepatobiliary, pancreatic, cardiothoracic, gynecologic, head and neck, orthopedic, emergency, and urologic subspecialties. Meta-analyses in these fields consistently demonstrate abbreviated hospital stays and decreased rates of extra-surgical infectious sequelae, such as pneumonia and urinary tract infections [14,15,16,17]. Additionally, these pathways are highly cost-effective, yielding a return on investment between 1.05 and 7.31, which translates to a net savings of up to $5898 per patient in colorectal cohorts [18]. The contrasting findings regarding SSI risk reduction across these varied surgical contexts underscore the necessity for the present narrative review.

## 2. Results

### 2.1. ERAS Protocols and SSIs in General Surgery

The impact of Enhanced Recovery After Surgery (ERAS) and Fast-Track Surgery (FTS) pathways on healthcare-associated infections (HAIs) was evaluated in a landmark systematic review and meta-analysis of randomized controlled trials (RCTs) by Grant et al. [14]. Focusing on open and laparoscopic abdominal and pelvic procedures, the authors isolated the incidence of primary HAIs after excluding studies with a high risk of bias. Their findings demonstrated a significant reduction in postoperative SSI risk within the ERAS/FTS cohorts compared to conventional management (Relative Risk [RR] = 0.61; 95% Confidence Interval [CI] = 0.41–0.90; *p* = 0.01; heterogeneity *p* = 0.84, I^2^ = 0%). A target subgroup analysis of colorectal surgeries confirmed this therapeutic trend, demonstrating a persistent decrease in SSI rates (RR = 0.73; 95% CI = 0.55–0.98; *p* = 0.04; heterogeneity *p* = 0.47, I^2^ = 0%).

Conversely, observational data have presented conflicting outcomes. A large-scale retrospective cohort study by Wang et al. [19] reported an overall baseline SSI incidence of 3.3% across a diverse general surgery population. The highest infection burden was isolated in gastrointestinal surgeries (8.72%), followed by hepatobiliary/pancreatic surgery (1.90%), herniorrhaphy (1.82%), and thyroid/breast procedures (0.46%). To control for baseline selection bias across these distinct specialties, a propensity score-matched analysis was performed. Following adjustment, SSI rates were 2.80% in the ERAS cohort and 3.98% in the non-ERAS cohort; however, this variance failed to achieve statistical significance (*p* = 0.534).

More recent literature continues to reflect these disparities. A recent cross-sectional study by [20] identified significantly lower SSI rates among patients managed via ERAS pathways compared to conventional cohorts (14.9% vs. 33.3%, *p* < 0.001) [20]. However, these findings require cautious interpretation due to several inherent methodological vulnerabilities: the cross-sectional design precludes causal inference, the reliance on convenience sampling introduces selection bias, and the utilization of self-reported data renders the endpoints vulnerable to recall and response biases.

To reconcile these conflicting data streams, Gillespie et al. [21] conducted a comprehensive umbrella review spanning literature published between 2010 and 2025. This review aggregated both RCTs and non-randomized designs involving adults undergoing major digestive procedures (including esophageal, gastric, hepatobiliary, pancreatic, and colorectal resections). The pooled meta-analysis for SSI endpoints—encompassing 11 systematic reviews and 42 unique primary RCTs (n = 5112)—yielded an estimated odds ratio (eOR) of 0.70 (95% CI = 0.59–0.82, *p* < 0.001). This indicates a statistically significant 30% reduction in SSI risk associated with global ERAS implementation. Notably, anatomical subgroup analyses revealed a pronounced 45% risk reduction specifically within abdominal surgeries, whereas other subspecialties demonstrated no statistically significant associations. Despite the strength of these pooled effect sizes, Gillespie et al. noted that the overall quality of evidence remained weak and inconclusive due to consistently low methodological quality scores across the primary literature. Substantial heterogeneity was introduced by wide variations in protocol composition (ranging from 5 to 28 discrete clinical elements), poorly defined “standard care” control arms that potentially overlapped with ERAS interventions, variations in clinician expertise, and institutional differences in absolute protocol adherence. The results of studies regarding specific surgical procedures are presented in Table 1.

### 2.2. Upper Gastrointestinal (GI) Surgery

The clinical evidence evaluating the specific impact of ERAS pathways on SSI incidence following upper GI surgery remains limited and inconclusive (Table 2). In a comparative cohort study, Wu et al. [28] observed no statistically significant reduction in SSI rates when comparing ERAS implementation to conventional care in patients undergoing major upper GI procedures, including gastrectomies, esophagectomies, and duodenal resections. This lack of detectable variance is mirrored in broader pooled analyses. A systematic review and meta-analysis of 14 randomized controlled trials (RCTs) involving 1515 patients undergoing gastric and colorectal resections evaluated standard care against perioperative management aligned with the ERAS Society guidelines (requiring a minimum implementation of eight discrete elements) [29]. The pooled analysis demonstrated no significant difference in SSI rates between the ERAS and conventional care cohorts (Relative Risk [RR] = 0.83; 95% Confidence Interval [CI] = 0.46–1.52; *p* = 0.56). Similarly, an updated meta-analysis by Liu et al. [30] focusing exclusively on oncological gastrectomies demonstrated that while ERAS pathways significantly mitigated overall postoperative morbidity, no independent or statistically significant reduction was achieved regarding SSIs. The investigators noted that the resulting confidence intervals were notably wide, rendering the pooled data compatible with both a modest relative clinical benefit and potential harm.

### 2.3. Lower Gastrointestinal (GI) Surgery

The data evaluating ERAS pathways within colorectal surgery present a compelling dichotomy between randomized and observational study designs (Table 3). A recent systematic review and meta-analysis of RCTs [31] failed to demonstrate a statistically significant reduction in SSI rates between patients managed via ERAS protocols versus conventional care (13.33% vs. 30.0%, *p* = 0.117), a finding likely confounded by limited sample sizes and insufficient statistical power to detect differences in secondary infectious endpoints. In contrast, multiple large-scale observational and retrospective cohort investigations strongly support a meaningful association between high ERAS compliance and mitigated SSI rates in colorectal resections [32,33,34,35]. However, these non-randomized designs are frequently vulnerable to confounding biases, including temporal shifts in neoadjuvant chemoradiation protocols, the evolution of specific ERAS elements over prolonged observation windows, and baseline disparities in patient risk profiles.

To isolate the compounding efficacy of these protocols, D’Souza et al. [36] investigated the sequential implementation of formal surgical site infection prevention bundles (SSIBs) alongside standardized ERAS pathways in a colorectal resection cohort. Comparing the baseline control group to the combined post-SSIB/post-ERAS cohort revealed a significant decrease in mean length of stay (7.6 vs. 5.5 days, *p* = 0.04), aggregate wound complications (14.7% vs. 6.5%, *p* = 0.049), and superficial incisional SSIs (8.2% vs. 1.8%, *p* = 0.047). A distinct downward trend was also observed for deep organ-space SSIs (7.3% vs. 4.7%, *p* = 0.40), though it did not achieve statistical significance.

Furthermore, the impact of ERAS has been validated in specific minor colorectal procedures. Madan et al. [37] conducted an RCT focused on patients undergoing elective ileostomy or colostomy reversal, explicitly excluding cases requiring formal midline laparotomies. The interventional ERAS cohort—characterized by the omission of mechanical bowel preparation, optimized goal-directed fluid therapy, and immediate postoperative oral intake—demonstrated a significantly lower incidence of SSIs compared to the standard care group (12.5% vs. 32.5%, *p* = 0.032).

### 2.4. Emergency Surgery

The overwhelming body of evidence investigating the application of ERAS pathways within acute care and emergency settings comprises randomized controlled trials (RCTs) and recent systematic reviews with meta-analyses. Although clinical presentations across these datasets vary, the predominant focus centers on patients presenting with gastrointestinal (GI) tract perforations requiring urgent laparotomy.

Pranavi et al. [38] conducted an open-label, superiority RCT explicitly targeting patients with GI perforation peritonitis, comparing a modified ERAS protocol against conventional standard care. The inclusion criteria captured patients requiring emergency laparotomy, while excluding high-risk individuals presenting with ASA 4E status, overt septic shock, localized peritonitis, or those requiring vasopressor or ventilatory support upon admission. The modified ERAS bundle was comprehensive, utilizing intraoperative goal-directed fluid therapy (GDFT), a specialized anesthetic protocol, an opioid-sparing multimodal analgesic strategy (relying on short-acting opioids strictly for rescue therapy), and accelerated mobilization and nutrition initiated on postoperative day (POD) 0. Their results demonstrated a profound reduction in SSI incidence, which fell from 58.0% in the conventional care group to 20.0% in the ERAS cohort (*p* < 0.001), with zero instances of fascial dehiscence or readmission reported in either arm.

Similarly, Sharma et al. [39] evaluated the efficacy of a 21-item emergency ERAS protocol in hemodynamically stable patients (ASA I–III) undergoing surgery for GI perforation or mechanical intestinal obstruction. Their pathway emphasized physiological preservation via aggressive normothermia maintenance, restrictive fluid models, and the omission of routine surgical drainage. The investigators reported a statistically significant decrease in major infectious complications, specifically pneumonia and SSIs, dropping from 61.2% in the conventional arm to 36.7% in the ERAS group (*p* = 0.015). However, the authors noted a potential age-related confounding variable, as the conventional care cohort was significantly older at baseline, which may have artificially inflated complication rates.

Several specialized RCTs have focused tightly on the subset of gastroduodenal perforations managed via omental (Graham) patch repair [40,41,42]. In an evaluation of 41 patients [40], investigators observed that while the ERAS cohort experienced a lower incidence of superficial incisional SSIs (10.5% vs. 18.2%), the variance failed to achieve statistical significance (*p* = 0.489), likely limited by the systematic exclusion of high-risk features such as multiple perforations or defects exceeding 1 cm in diameter. Another RCT [41] analyzing 64 patients implemented a “t-ERAS” protocol incorporating thoracic epidural analgesia and early surgical drain removal. Although the t-ERAS group demonstrated an absolute downward trend in both superficial (12.0% vs. 20.0%) and organ-space SSIs (4.0% vs. 12.0%), these findings were not statistically significant (*p* = 0.70 and *p* = 0.60, respectively).

Furthermore, the ERASE Trial [42] investigated 60 patients and reinforced the safety profile of accelerated pathways. The SSI rate within the conventional arm (33.3%) was double that of the ERAS cohort (16.7%), though it did not reach mathematical significance (*p* = 0.170). Crucially, the ERASE trialists highlighted an implementation barrier: while postoperative protocol items achieved near 100% compliance, intraoperative fluid balance targets were successfully met in only 10% of cases.

To aggregate these localized data streams, Zeyara et al. [43] performed a systematic review and meta-analysis of six acute care RCTs (n = 356) restricted to perforated peptic ulcers. Their synthesis confirmed that fast-track pathways significantly reduced the risk of both superficial incisional SSIs (risk difference = −0.12, *p* = 0.002) and deep/organ-space SSIs (risk difference = −0.03, *p* = 0.032). The authors cautioned, however, that these metrics may reflect geographic bias, as all pooled studies were conducted within Asian populations, and high-risk patients (ASA greater than 3, large or malignant perforations) were universally excluded.

Beyond gastroduodenal pathology, Pawar et al. [44] evaluated 78 patients presenting with acute abdomen (predominantly small bowel obstructions), excluding vascular emergencies and peptic ulcerations. While the ERAS group exhibited fewer overall complications, the relative drops in superficial (5.13% vs. 12.82%) and deep SSIs (0.00% vs. 5.12%) missed statistical significance, a finding highly indicative of a Type II error due to an underpowered sample size for secondary infectious outcomes.

Finally, the application of ERAS within emergency abdominal trauma has been assessed in a recent RCT [45] capturing patients with hollow viscus and solid organ injuries from both blunt and penetrating mechanisms. Interestingly, formal randomization occurred 24 h postoperatively, restricting the intervention exclusively to the postoperative phase. Despite this delayed initiation, the study demonstrated a significant reduction in deep organ-space SSIs, dropping from 24.0% in the control group to 0.0% in the ERAS arm (*p* = 0.030), suggesting that even isolated postoperative adherence to ERAS principles yields substantial clinical dividends in trauma populations. The current body of evidence suggests that ERAS protocols in emergency surgery are not only feasible but are consistently associated with a downward trend in SSI incidence and accelerated functional recovery. However, several systematic limitations must be considered. First, most literature explicitly excludes high-risk patients (ASA III–IV, overt septic shock, or severe multi-trauma), meaning the therapeutic benefits for the critically ill demographic remain unproven. Second, there is a notable paucity of data regarding laparoscopic emergency procedures, as the current literature focuses almost exclusively on open laparotomy cohorts. Third, many individual RCTs remain severely underpowered to detect statistically significant differences in secondary outcomes such as SSIs. Finally, as underscored by the ERASE trial, achieving complete compliance with intraoperative pathway elements remains an arduous logistical challenge in emergency settings compared to elective surgical workflows. The details of all included studies are presented in Table 4.

## 3. Discussion

The findings of this review underscore the transformative impact of Enhanced Recovery After Surgery (ERAS) on global postoperative outcomes, with critical implications for the mitigation of SSIs. While ERAS was originally pioneered to optimize physiological recovery and decrease length of stay, its role as a complementary mechanism for infection control is becoming increasingly evident across both elective and emergency surgical landscapes. The aggregated evidence indicates that ERAS functions as a comprehensive perioperative ecosystem that may lower SSI risk through several indirect, synergistic mechanisms in different surgical settings. Reduction in surgical stress, maintenance of normothermia and euvolemia, earlier mobilization, earlier feeding, avoidance or early removal of tubes, and higher protocol compliance are the crucial points of ERAS in reducing SSIs. Crucially, these key ERAS components directly intersect with established, independent SSI prevention bundles promulgated by global health organizations, including the World Health Organization (WHO), the Centers for Disease Control and Prevention (CDC), and the National Institute for Health and Care Excellence (NICE) [2,46,47]. Despite the biological plausibility of this intersection, the global body of evidence evaluating the direct relationship between ERAS pathways and isolated SSI reduction must currently be classified as low to very low in methodological quality. While the literature supports a robust, reproducible effect of ERAS on accelerating recovery kinetics, its independent efficacy in preventing SSIs varies dramatically based on procedural complexity, baseline patient risk profiles, absolute protocol compliance, and whether explicit, infection-specific bundle elements are concurrently incorporated.

Despite these methodological limitations, this clinical synergy is particularly pronounced within emergency surgery workflows, where baseline SSI incidence is inherently higher, and in laparoscopic cholecystectomy. Accumulating data also suggest a positive trend toward SSI reduction in bariatric surgery, hepatobiliary resections, liver transplantation, and pulmonary resections; however, these conclusions remain primarily derived from retrospective, historical cohort observations.

### Limitations

Despite these positive trends, the “weak and inconclusive” classification of some evidence by umbrella reviews [21] indicates the need for more standardized research. The primary challenges identified include the lack of uniformity in which items constitute an ERAS protocol, making direct comparisons between centers difficult, inconsistent reporting of adherence to the ERAS protocol items, overlap of certain ERAS items with SSI prevention bundle items, and lack of a universal definition of SSIs. Moreover, as ERAS becomes the new gold standard, “conventional care” groups in modern trials often inadvertently adopt ERAS elements, which can dilute the statistical significance of comparative studies, particularly observational studies comparing pre- and post-ERAS periods.

A critical conceptual challenge in ERAS literature is determining whether high protocol compliance drives superior outcomes, or if it merely serves as a proxy for a healthier baseline patient status (also known as “the healthy performer bias”). Critics frequently argue that frailer patients or those with severe comorbidities are inherently less capable of tolerating early mobilization or aggressive feeding, leading to early protocol discontinuation and an artificial inflation of success within high-adherence cohorts. However, recent advanced analytics deploying explainable machine learning models to decouple baseline patient risk from protocol adherence have demonstrated that high ERAS compliance exerts an independent, protective therapeutic effect that persists regardless of baseline physical status [48].

Another frequent point of contention in perioperative implementation science is the management of patient cohorts with pre-existing diabetes mellitus within ERAS frameworks. Because perioperative hyperglycemia is a well-established, independent driver of microvascular dysfunction, impaired neutrophil phagocytosis, and subsequent SSI pathogenesis, critics often hypothesize that the strict exclusion of diabetic patients from ERAS pathways—particularly regarding the utilization of preoperative carbohydrate loading (CHO)—artificially biases comparative studies by leaving higher-risk patients in the conventional care arm. However, contemporary clinical guidelines explicitly state that diabetes mellitus is not a contraindication to ERAS enrollment, nor does it necessitate protocol exclusion. Instead, modern pathways advocate for a risk-stratified, modified approach to preoperative metabolic optimization, based on preoperative HbA1c levels: patients with well-controlled or moderately controlled diabetes (typically defined as a baseline HbA1c up to 8.0%) safely tolerate standard or volume-adjusted preoperative CHO beverages without experiencing detrimental glycemic excursions, provided their home medications are appropriately adjusted. For insulin-dependent or poorly optimized individuals, the administration of preoperative CHO is frequently coupled with tailored, low-dose basal insulin or continuous intravenous insulin infusions to maintain strict perioperative euglycemia.

As noted by some researchers [12], minimally invasive surgery (MIS) is a powerful, independent protector against SSIs. Disentangling the specific benefits of the ERAS pathway from those of MIS remains a complex task for future research.

## 4. Materials and Methods

A comprehensive literature search was conducted across the PubMed, Scopus, and Embase databases spanning from January 2016 to March 2026 using the following search strategy: (“Enhanced Recovery After Surgery”[Mesh] OR “Enhanced Recovery After Surgery”[Title/Abstract] OR “ERAS”[Title/Abstract]) AND (“Surgical Wound Infection”[Mesh] OR “Surgical Site Infection”[Title/Abstract] OR “Surgical Site Infections”[Title/Abstract] OR “SSI”[Title/Abstract]). The initial search retrieved 145 unique titles that were independently screened for inclusion by four authors (LP, PC, SS, and MB). Discrepancies during the screening process were resolved by consensus. Studies were evaluated based on pre-specified exclusion criteria: duplicates, studies in populations other than adults, studies in languages other than English, studies with inappropriate comparison groups or not reporting the endpoint of interest (SSI rates), and studies that had been formally retracted by the publishing journals due to data integrity issues or other editorial concerns. Following the application of these criteria, 23 articles met all eligibility requirements. To ensure maximum comprehensiveness, a manual “snowballing” search strategy was executed by cross-referencing the bibliographies of all initially included studies. This secondary cross-referencing process identified six additional relevant articles, resulting in a final total of 29 studies included for narrative synthesis in this review.

## 5. Conclusions

ERAS pathways consistently improve postoperative recovery and reduce LOS across multiple surgical settings; however, their independent, isolated effect on the incidence of SSIs is less clear. Current evidence suggests that ERAS frameworks contribute to SSI mitigation by standardizing perioperative care pathways and systematically attenuating modifiable, host-specific physiological risk factors. Nevertheless, this protective effect is heavily modulated by absolute protocol adherence, the chosen surgical access modality, and the concurrent utilization of procedure-specific infection prevention bundles.

Consequently, ERAS should be conceptualized as a powerful, complementary clinical framework for infection prevention rather than a substitute for established, dedicated SSI prevention bundles. The most defensible conclusion is that ERAS pathways significantly reduce SSI risk only when executed with high fidelity and paired deliberately with evidence-based infection prevention strategies. To resolve remaining controversies, future clinical trials must utilize standardized SSI classification systems, report granular element-by-element protocol compliance, explicitly differentiate superficial incisional from deep and organ-space infections, and deploy multivariate analyses to isolate the specific ERAS components most strongly correlated with infection reduction.

## Figures and Tables

**Table 1:** ERAS and SSIs—specific surgical procedures.

Year [Ref.]	Study Type	Patient/Population	Intervention/Comparison	Outcome: SSIs
2018 [17]	RS	Pancreatic surgery	ERAS vs. conventional care	26% vs. 27% (*p* = ns). higher occurrence of SSIs (67% vs. 6%; *p* < 0.001) for ERAS compliance < 70%
2025 [22]	RS	Bariatric surgery	Pre- vs. Post-ERAS implementation	superficial SSIs 0.99% vs. 0.11%; *p* = 0.021
2024 [23]	RS	Liver Transplantation	Pre- vs. Post-ERAS implementation	37.5% vs. 5%; *p*= 0.001
2024 [24]	RS	Complex spine surgery	Pre- vs. Post-ERAS implementation	0.6% vs. 0.3%; *p*= 1.376
2024 [25]	PS	Pulmonary resection	Pre- vs. Post-ERAS implementation	1.6% vs. 0.2%; *p* = 0.04
2025 [26]	RS/PSMA	robotic nephrectomy	Pre- vs. Post-ERAS implementation	1% vs. 0%; *p* = 0.317
2025 [27]	RCT	laparoscopic cholecystectomy	ERAS vs. conventional care	2% vs. 12%; *p* = 0.046

RS: retrospective study; PS: prospective study; PSMA: propensity score-matching analysis; RCT: randomized controlled trial; ERAS: Enhanced Recovery After Surgery; SSIs: Surgical Site Infections.

**Table 2:** ERAS and SSIs—Upper GI surgery.

Year [Ref.]	Study Type	Patient/Population	Intervention/Comparison	Outcome: SSIs
2024 [28]	SR/MA	gastrectomy, esophagectomy and duodenectomy	ERAS vs. conventional care	OR 0.80 (95% CI: 0.39–1.64, *p* = 0.542)
2023 [29]	SR/MA of RCT	Gastrectomy and colorectal surgery	ERAS vs. conventional care	No significant differences
2025 [30]	SR/MA of RCT	oncological gastrectomy/gastric resection	ERAS vs. conventional care	OR 0.66 (0.22–1.95)

SR/MA: systematic review/metanalysis; RCT: randomized controlled trial; ERAS: Enhanced Recovery After Surgery.

**Table 3:** ERAS and SSIs—Lower GI Surgery.

Year [Ref.]	Study Type	Patient/Population	Intervention/Comparison	Outcome: SSIs
2025 [31]	SR/MA of RCT	colorectal surgery	ERAS vs. conventional care	13.33% vs. 30.0%; *p* = 0.117
2021 [32]	RS	elective colorectal and gynecologic/oncology procedures	ERAS	SSI 3.3%
2019 [33]	RS	Colorectal surgery	Pre- vs. Post-ERAS implementation	12.26% vs. 5.04%; *p* = 0.004
2021 [34]	QES	Colorectal cancer surgery	ERAS vs. conventional care	7.5% vs. 15.8%; *p* = 0.031
2021 [35]	RS	Rectal cancer surgery	Pre- vs. Post-ERAS implementation	24% vs. 5%; *p* = 0.013
2019 [36]	RS/PSMA	Colorectal surgery	pre-ERAS/pre-SSIB (control group) vs. post-ERAS/post-SSIB	superficial SSIs 8.2% vs. 1.8%; *p* = 0.047
2023 [37]	RCT	elective stoma reversal	ERAS vs. conventional care	12.5% vs. 32.5%; *p* = 0.032

SR/MA: systematic review/metanalysis; RCT: randomized controlled trial; RS: retrospective study; QES: quasi-experimental study; PSMA: propensity score-matching analysis; ERAS: Enhanced Recovery After Surgery; SSIs: Surgical Site Infections; SSIB: Surgical Site Infection prevention bundle.

**Table 4:** ERAS and SSIs—Emergency surgery.

Year [Ref.]	Study Type	Patient/Population	Intervention/Comparison	Outcome: SSIs
2022 [38]	RCT	GI perforation.	ERAS vs. standard of care	20% vs. 58%; *p* < 0.001
2021 [39]	RCT	GI perforation or intestinal obstruction	ERAS vs. standard of care	36.7% vs. 61.2%; *p* = 0.015
2023 [40]	RCT	perforated duodenal ulcer	ERAS vs. standard of care	superficial SSIs: 10.5% vs. 18.2%; *p* = 0.489 deep SSIs: 0–4.5%; *p* = 0.347
2023 [41]	RCT	GI perforation	ERAS vs. standard of care	superficial SSIs: 12% vs. 20%; *p* = 0.7 organ-space SSIs: 4% vs. 12%; *p* = 0.6
2025 [42]	RCT	GI perforation	ERAS vs. standard of care	16.66% vs. 33,33%; *p* = 0.17
2024 [43]	SR-MA	GI perforation	ERAS vs. standard of care	superficial SSIs: Risk Difference −0.12; *p* = 0.002 deep SSIs: Risk Difference −0.03; *p* = 0.032
2025 [44]	RCT	acute abdomen	ERAS vs. standard of care	superficial SSIs: 5.13% vs. 12.82%; *p* = 0.181 deep SSIs: 0% vs. 5.12%; *p* = 0.152
2024 [45]	RCT	abdominal trauma	ERAS vs. standard of care	superficial SSIs: 12% vs. 8%; *p* = 1 deep SSIs: 0% vs. 24%; *p* = 0.03

RCT: randomized controlled trial; SR-MA: systematic review/metanalysis; GI: gastrointestinal: ERAS: Enhanced Recovery After Surgery; SSIs: Surgical Site Infections.

## Data Availability

No new data were created or analyzed in this study.
